# Combined Microencapsulated Islet Transplantation and Revascularization of Aortorenal Bypass in a Diabetic Nephropathy Rat Model

**DOI:** 10.1155/2016/9706321

**Published:** 2016-03-28

**Authors:** Yunqiang He, Ziqiang Xu, Hongxing Fu, Bin Chen, Silu Wang, Bicheng Chen, Mengtao Zhou, Yong Cai

**Affiliations:** ^1^Zhejiang Provincial Top Key Discipline in Surgery, Wenzhou Key Laboratory of Surgery, Department of Surgery, The First Affiliated Hospital, Wenzhou Medical University, Wenzhou, Zhejiang 325000, China; ^2^Department of Transplantation, The First Affiliated Hospital, Wenzhou Medical University, Wenzhou, Zhejiang 325000, China; ^3^School of Pharmacy, Wenzhou Medical University, Wenzhou, Zhejiang 325000, China; ^4^Department of B-Mode Ultrasound, The First Affiliated Hospital, Wenzhou Medical University, Wenzhou, Zhejiang 325000, China

## Abstract

*Objective.* Revascularization of aortorenal bypass is a preferred technique for renal artery stenosis (RAS) in diabetic nephropathy (DN) patients. Restenosis of graft vessels also should be considered in patients lacking good control of blood glucose. In this study, we explored a combined strategy to prevent the recurrence of RAS in the DN rat model.* Methods.* A model of DN was established by intraperitoneal injection of streptozotocin. Rats were divided into 4 groups: SR group, MIT group, Com group, and the untreated group. The levels of blood glucose and urine protein were measured, and changes in renal pathology were observed. The expression of monocyte chemoattractant protein-1 (MCP-1) in graft vessels was assessed by immunohistochemical staining. Histopathological staining was performed to assess the pathological changes of glomeruli and tubules.* Results.* The levels of urine protein and the expression of MCP-1 in graft vessels were decreased after islet transplantation. The injury of glomerular basement membrane and podocytes was significantly ameliorated.* Conclusions.* The combined strategy of revascularization and microencapsulated islet transplantation had multiple protective effects on diabetic nephropathy, including preventing atherosclerosis in the graft vessels and alleviating injury to the glomerular filtration barrier. This combined strategy may be helpful for DN patients with RAS.

## 1. Introduction

Long-term hyperglycemia not only significantly impairs the microstructure of the kidney, leading to diabetic nephropathy (DN), but also causes renal artery stenosis (RAS) in diabetic patients [1, 2]. The RAS detection rate in type 2 diabetes patients was recently reported to be 36.9% using duplex ultrasound scanning [3]. Progression of RAS to occlusion leads to uncontrolled hypertension, progressive renal hypofunction, and even renal failure [4]. One clinical study has shown that the presence of RAS in type 2 diabetes patients raises the relative risk of progressing to renal failure 2.4-fold compared with patients without RAS [5].

Surgical revascularization of renal arteries has been considered as a primary maneuver to restore blood flow and improve renal function in diabetic patients with RAS [6, 7]. However, a high percentage of restenosis in aortorenal bypass has been shown in diabetic patients with poorly controlled blood glucose [3, 8]. Due to poor blood glucose control, separate revascularization of renal arteries cannot provide effective long-term protection of renal function, which leads to atherosclerosis and restenosis of the transplanted blood vessels [3]. Therefore, it is necessary to supply a comprehensive and effective treatment for DN patients undergoing RAS.

Microencapsulated islet transplantation (MIT) has been verified as an effective therapy for diabetes mellitus. Microencapsulation can reduce the rejection response in the absence of immunosuppression [9–11]. Islets are enveloped within a semipermeable membrane that allows the insulin and glucose to pass freely; however, it prevents the entry of immune-active cells and molecules [11, 12]. It has been shown in different strains of diabetic rodents that MIT can maintain blood glucose in the normal range and alleviate damage to the kidneys [13]. Furthermore, according to several reports, MIT could ameliorate or even reverse the damage in the kidney of DN rats [14]. Thus, MIT is a useful strategy to control blood glucose and alleviate damage to the kidney for either diabetic or DN rodents.

In this study, we combined the revascularization of the renal artery and microencapsulated islet transplantation in diabetic rats. The aims were to observe the impact of microencapsulated islet transplantation on pathological changes in the bypass vessels and the glomerular filtration barrier and to explore the possibility of the clinical applications of this approach in diabetic nephropathy patients undergoing RAS.

## 2. Materials and Methods

### 2.1. Animals

Thirty male Sprague-Dawley (SD) rats and twenty-four male Wistar rats at 8 weeks of age (180–220 g) were obtained from the Laboratory Animal Centre of Wenzhou Medical University. Wistar rats were used as islet donors. All animal procedures were based on international guidelines and approved by the Wenzhou Medical University Animal Policy and Welfare Committee. Twenty SD rats received a single intraperitoneal dose of streptozotocin (Sigma-Aldrich, USA, 55 mg/kg) to establish diabetes. One week later, blood samples from the tail vein were used to determine the blood glucose levels. Rats were considered to be diabetic if the blood glucose levels were between 16 mmol/L and 30 mmol/L on more than two consecutive days without fasting [15]. At 8 weeks after diabetes induction, the urine protein-to-creatinine ratio and microalbumin-to-creatinine ratio (ACR) were determined, and TEM detection was performed to assess whether rat diabetic nephropathy models had been successfully established [16]. The rats were divided into 4 groups. The first group (*n* = 4) was treated by surgical revascularization of renal arteries (SR group); the second group (*n* = 4) was treated by microencapsulated islet transplantation (MIT group); the third group (*n* = 4) was treated by a combination of microencapsulated islet transplantation and surgical revascularization (Com group); and the fourth group (*n* = 6) consisted of untreated diabetic nephropathy rats (DN group).

### 2.2. Revascularization of Rat Aortorenal Bypass

**Figure 1:**
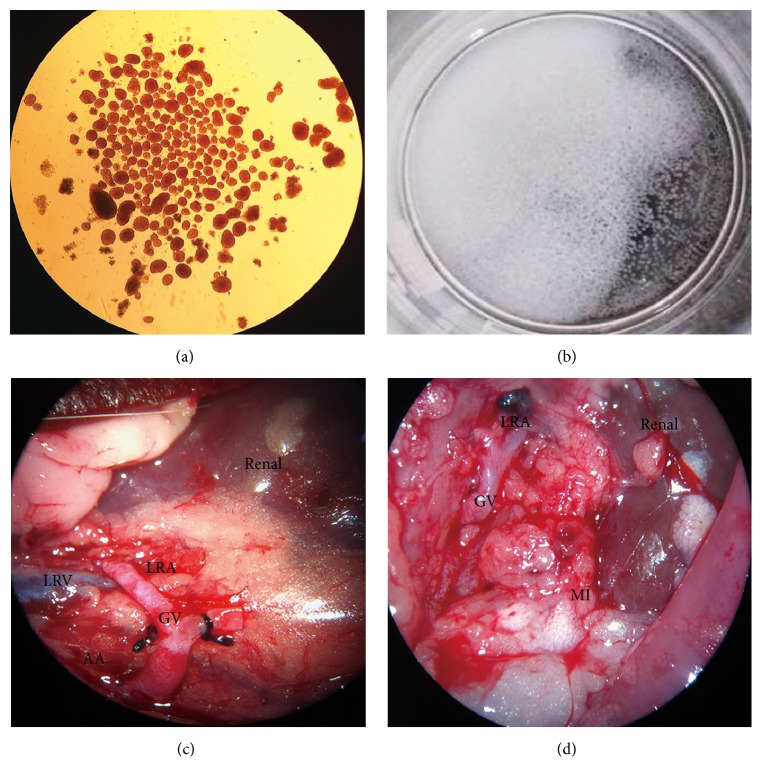
Microencapsulated islet transplantation and revascularization of aortorenal bypass. (a) Isolated and purified islets (×40). (b) Microencapsulation of islets. (c) Vascular reconstruction between left renal artery and abdominal aorta. (d) The vascular graft had blood flow, and microcapsules were distributed in the abdominal cavity and wall in the Com group (4 weeks after transplantation). LRA, left renal artery; LRV, left renal vein; GV, graft vessel; AA, abdominal aorta; MI, microencapsulated islets.

SD rats were anesthetized with pentobarbitone (70 mg/kg). An abdominal longitudinal incision was made to dissociate the lower abdominal aorta and bilateral external iliac artery. The unilateral external iliac artery with fewer branches was dissociated for approximately 0.5 cm, while ligation was performed for the opposite external iliac artery at the branches of the aorta. Nearly 1.5 cm of the abdominal aorta and 0.5 cm of the extended external iliac artery were incised, respectively. Specimens were submerged in saline for use during grafting. After successful anesthetization of rats with diabetic nephropathy, a longitudinal incision was made in the upper abdomen. Bowels and stomach were pushed to the right side to expose the left renal blood vessels. With the aid of a microscope, the renal artery was carefully dissociated from behind left renal veins, and the abdominal aorta was partially dissociated by dragging a wire. Both ends of the renal arteries were occluded with vascular clamps, and ice was left around the kidney. An incision approximately 1 mm long was made on the renal artery. An end-to-side anastomosis between the donor's iliac vessels and renal arteries was performed using a microprobe. Then, an incision nearly 3 mm long was made on both ends of the abdominal aorta for occlusion, so that an end-to-side anastomosis could be performed between the abdominal aorta of the donor's blood vessels and the abdominal aorta of the acceptor. Next, vascular clamps were unloosed to locally increase pressure and stop bleeding. After 5 minutes of observation, no significant anastomotic bleeding was observed, and the kidney was restored to its normal color (Figure 1(c)). Following the operation, Ceftazidime (100 mg/kg) was hypodermically injected for 3 consecutive days.

### 2.3. Islets Encapsulation and Transplantation

Donor islets from three Wistar rats were transplanted into each recipient. Islet isolation was performed according to methods described previously [17, 18]. Briefly, islets were harvested by reverse perfusion of collagenase V into the common bile duct and purified by Histopaque (Sigma-Aldrich, USA) density gradient, followed by handpicking. The purified islets were incubated overnight in cell culture medium with 10% fetal bovine serum at 37°C, 5% CO_2_ condition. Total islet equivalents (IEQ) were calculated according to the islet equivalents calculation formula. Approximately 2500–3000 IEQ islets from three donors were used for each recipient. Islets were encapsulated in alginate-polylysine-alginate (APA) microcapsules, as described previously [19]. Alginate (Shanghai Analysis Chemical and Technology Co., Ltd., Shanghai, China) beads with rat islets were obtained using an electrostatic beads generator and fell into a solution of crosslinking ions. After washing with saline, these liquid droplets were coated with 0.15% alginate for 5 min and washed again with saline to generate the APA beads. The APA beads were dispersed using 55 mM sodium citrate to liquefy the central core. Finally, the microcapsules with islets were cultured overnight in Roswell Park Memorial Institute culture medium (RPMI-1640 Genome Biological Pharmaceutical Technology Co., Ltd., Hangzhou, China) for transplantation. The diameter of the microcapsules was approximately 500–600 *µ*m (Figure 1(b)). Before transplantation, the rats were anesthetized with pentobarbitone (70 mg/kg). Microcapsules with islets were injected into the peritoneal cavity using a 20-gauge catheter.

### 2.4. Specimen Collection

Blood glucose levels of all groups were measured twice a week throughout the experimental period. The 24 h urine and random urine specimens of rats were collected using metabolic cages at the end of the study and determined with a Fully Automatic Biochemistry Analyzer (Hitachi 7600, Japan). All rats were humanely sacrificed after 4 weeks. The kidney was perfused in situ with saline by aortic cannulation. Graft vessels were transferred into 4% paraformaldehyde and a part of the renal tissue was removed into 2.5% glutaraldehyde for transmission electron microscopy analysis.

### 2.5. Histopathology and Immunohistochemistry

Renal and vessel tissues were immersion-fixed in 4% paraformaldehyde for 48 h, embedded in paraffin, and cut into 4 *μ*m slices. After deparaffinization and rehydration, the sections were stained separately with hematoxylin-eosin (HE) staining and periodic acid-Schiff (PAS) staining to observe the pathological changes in glomeruli and tubules by microscope. Monocyte chemoattractant protein-1 (MCP-1) protein is a marker of arteriosclerosis and can be detected early in intimal hyperplasia [20]. The expression of MCP-1 was detected by immunohistochemical staining with rabbit polyclonal MCP-1 (Beijing Biosynthesis Biotechnology Co., China). Briefly, graft vessels were sliced into 4 *μ*m sections. After removal of the paraffin by xylene and dehydration by graded alcohol, slides were transferred into a 10 mmol/L citrate buffer solution and heated at 100°C for 10 minutes for antigen retrieval. After washing, 3.0% peroxide was applied for 10 minutes to block the activity of endogenous peroxidase. To avoid nonspecific staining, slides were incubated with normal goat serum at room temperature for 60 minutes. The primary anti-rabbit MCP-1 polyclonal antibody was diluted 1 : 150 and incubated with the section at 4°C overnight. Negative control sections were stained under the identical conditions by substituting the primary antibody with equivalent concentrations of normal rabbit IgG. Tissue sections were washed with phosphate-buffered saline (PBS, 0.01 M), incubated with secondary antibodies, and visualized with diaminobenzidine (DAB, brown color, ZSGB-BIO, Beijing, China) and hematoxylin counterstaining. To evaluate MCP-1 staining, five fields for each vessel slice were scored semiquantitatively using the Image-Pro Plus system (Media Cybernetics, Silver Spring, MD).

### 2.6. Electron Microscopy Examination

Small pieces of renal cortical tissue from each group were processed according to a previously described method [21] and examined using a transmission electron microscope (TEM; H-7700, Hitachi, Japan).

### 2.7. Statistical Analysis

Data were presented as the means ± standard error of the mean (SEM). Statistical analyses were performed using one-way analysis of variance, and *P* < 0.05 was considered significant. SPSS software (version 13.0) was used for statistical analyses.

## 3. Results

### 3.1. Model Building

The urine protein-to-creatinine ratio and ACR were determined to measure renal injury, and TEM detection of the kidney tissue was performed to observe pathological changes. The DN rats were considered a good model as they displayed local GBM thickening, mesangial expansion, podocyte depletion with fusion of foot processes, and abnormal glomerular filtration barrier structures. In addition, the mean values of urine protein-to-creatinine ratio and ACR were significantly higher compared to normal rats (*P* < 0.05). Ten DN model rats were used to perform revascularization of aortorenal bypass. The mean operative duration of surgical revascularization was 100.5 ± 16.5 min. It took less than 30 minutes to block the renal artery and abdominal aorta. Abdominal longitudinal incision was performed to observe the graft two days after the surgery. The patency of the graft vessel was defined as a sign of a successful operation (Figure 1(c)). In this study, the success percentage was 80% (8/10). Half of the rats with successful revascularization received the islet transplantation. Four weeks after revascularization, the blood flow at both proximal and distal anastomotic sites was observed, and the patency of the graft vessel was also shown in the study (Figure 1(d)). The results suggested that revascularization by aortorenal bypass was a feasible way to restore the renal blood flow.

### 3.2. Blood and Urine Parameters

**Figure 2:**
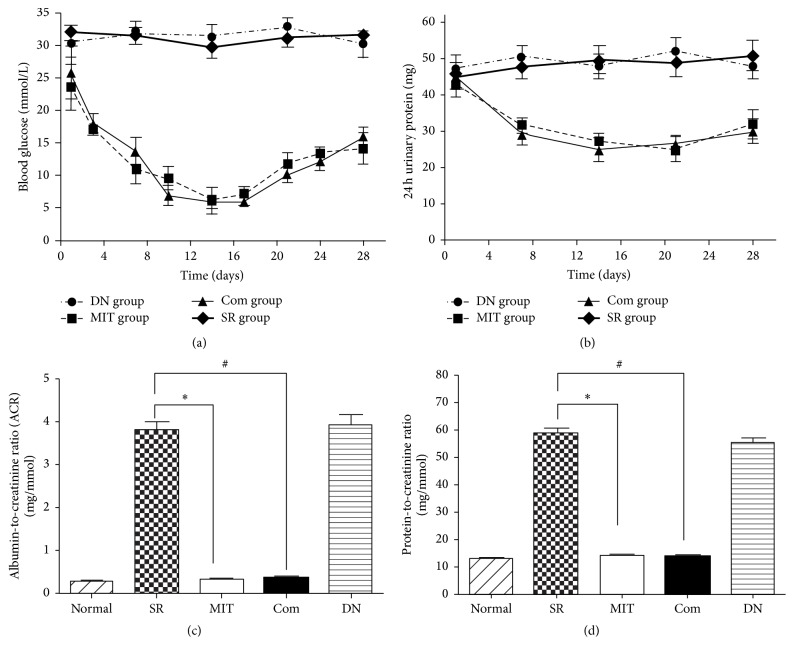
Blood glucose levels and urine analysis. After microencapsulated islet transplantation, the blood glucose level (a) and 24-hour urinary protein (b) were decreased significantly. ^*∗*^ *P* < 0.01, MIT group compared with the SR group; ^#^ *P* < 0.01, Com group compared with the SR group. (c) Urine albumin-to-creatinine ratio in each group; ^*∗*^ *P* < 0.001 and ^#^ *P* < 0.001. (d) Urine protein-to-creatinine ratio in each group; ^*∗*^ *P* < 0.001 and ^#^ *P* < 0.001.

As shown in Figure 2(a), the blood glucose levels reduced in both the MIT group and the Com group after microencapsulated islet transplantation, compared to the DN and SR groups. Meanwhile, 24-hour urine protein, random urine protein-to-creatinine ratio, and ACR in MIT group and Com group decreased significantly compared with the DN and SR groups (Figures 2(b)–2(d)). However, there were no significant differences between the MIT group and the Com group. Therefore, MIT could restore blood glucose to the normal range and ameliorate the renal damage in DN rats.

### 3.3. Change in the Glomerular Filtration Membrane

**Figure 3:**
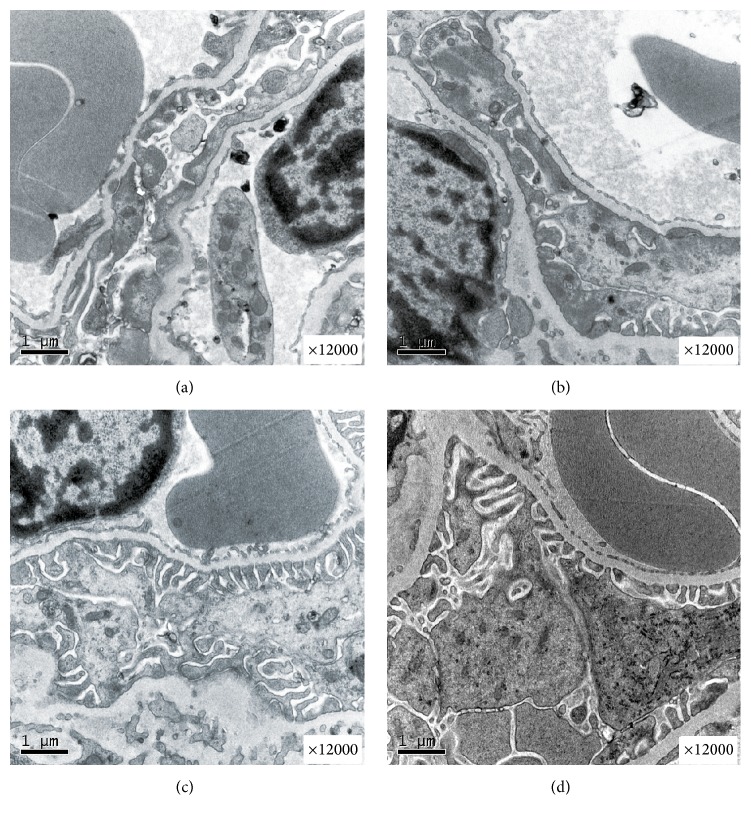
Electron microscopy. Irregular thickening of the GBM and the fusion of podocytes were observed in the DN group (a) and the SR group (b) by electron microscope. The fusion of podocytes foot processes was rare, and the thickening of the GBM was alleviated in the MIT group (c) and the Com group (d).

Irregular thickening of the glomerular basement membrane (GBM) and podocyte depletion with foot process fusion were observed in the DN group by transmission electron microscope (Figure 3(a)). At four weeks after MIT, the fusion of podocyte foot process and damage of the GBM were significantly improved in both the MIT group and the Com group (Figures 3(c) and 3(d)), but there was no improvement in the SR group (Figure 3(b)). This supports the conclusion that MIT ameliorates the damage to the GBM and to the podocytes in diabetic nephropathy rats.

### 3.4. Histopathology of Renal Tissues and Bypass Vessels

**Figure 4:**
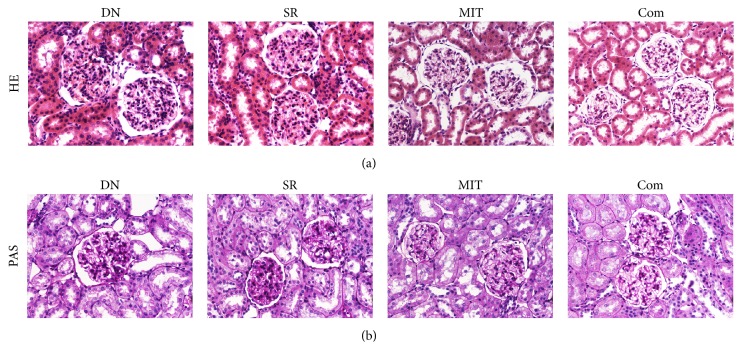
Histopathological staining of the renal tissues (original magnification ×400). HE staining (a) and PAS staining (b) were performed to observe the pathological changes in glomeruli and tubules in each group.

**Figure 5:**
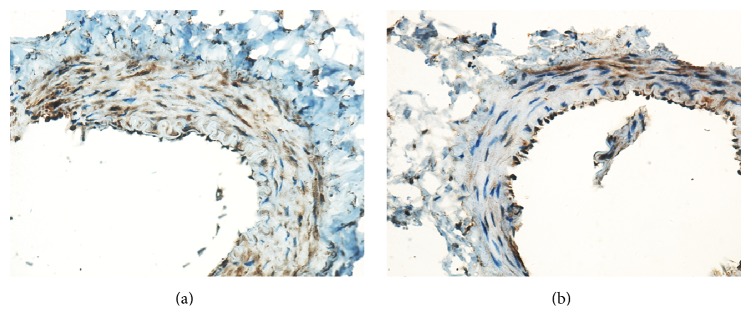
Immunohistochemical staining for MCP-1 in the graft vessels (original magnification ×400). (a) Surgical revascularization group: the expression of MCP-1 (brown staining of the granules) was obvious in the graft vessels wall. (b) Combined treatment group: the positive expression in the graft vessels was significantly decreased after islet transplantation.

The results of HE staining and PAS staining revealed that there were significant differences between the groups. Glomerular hypertrophy, thickening of tubular basement membrane, and hyperplasia of mesangial, glomerular, and tubular cells were obvious in the DN and SR groups as shown in the HE staining (Figure 4(a)). Also, the adhesion of glomerular capillaries and glycogen deposition in glomeruli and tubules were more significant in the DN and SR groups when compared with the MIT and Com groups (PAS staining, Figure 4(b)). The intimal hyperplasia of the bypass vessel by histological examination was not obvious after 4 weeks in the rats without islet transplantation. The short time of renal artery revascularization in this study makes it difficult to observe pathological difference in the bypass vessel. MCP-1 immunohistochemical staining was observed in graft vessels (Figure 5). Compared with the SR group (Figure 5(a)), the expression of MCP-1 decreased significantly in the Com group (Figure 5(b)). The mean of IOD/area (integrated optical density/area) in the Com group (0.215 ± 0.009) was significantly lower than that in the SR group (0.255 ± 0.009, *P* < 0.05). These data suggest that microencapsulated islet transplantation attenuates the atherosclerosis of the bypass vessel in diabetic nephropathy model rats.

## 4. Discussion

In this study, we combined the use of microencapsulated islet transplantation and revascularization of the renal artery to investigate the impact of microencapsulated islet transplantation on pathological changes in the bypass vessels and the glomerular filtration barrier. The results demonstrated that blood glucose levels could be maintained in the normal range after islet transplantation and that 24-hour urine protein, random urine microalbumin-to-creatinine ratio, and ACR significantly decreased when compared with nontransplanted diabetic nephropathy rats. In the later period of the experiment, the blood glucose levels of the MIT and Com groups slightly increased with the decreased secretory function of a small part of transplanted islets. However, the levels of blood glucose in the MIT and Com groups were still significantly lower than those in the SR and DN groups. TEM detection and immunohistochemical staining of graft vessels also revealed that pathological damage, such as thickening of the GBM and podocyte injury, and the expression of MCP-1 were alleviated after transplantation. These data show that islet transplantation can significantly improve blood glucose control, reduce renal injury, and protect graft vessels.

Long-term hyperglycemia is known to be a major cause of RAS, and once formed even the restoration of blood glucose control cannot suppress RAS progression [8]. Revascularization is known to be an effective treatment for RAS [22]. However, the effects of separate surgical revascularization of renal arteries on patients with diabetic nephropathy were unsatisfactory in the clinical treatment [5]. Under conditions of sustained high blood glucose, the restenosis of graft vessels was considerable. Hence, the control of high blood glucose was critical in the treatment of RAS in diabetic nephropathy.

Microencapsulated islet transplantation has become a promising treatment for severely diabetic patients to control hyperglycemia and prevent diabetic complications [23]. Several decades ago, studies found that high blood glucose in diabetic animal models could be returned to the normal range by transplantation of islets encapsulated with a semipermeable membrane. In our study, we encapsulated the islets with APA microcapsules, which protect the inner islets from mechanical stress and the immune system in recipients, while allowing bidirectional diffusion of oxygen, nutrients, hormones, glucose, and wastes [24]. This approach can reduce or prevent immunological rejection of the transplanted tissue in recipients without the need for immunosuppressants. Because the blood glucose levels were well controlled after islet transplantation, the protective effects on the graft vessels after revascularization were clear in our study.

Due to the short duration of renal artery revascularization in this study, it was hard to find atherosclerosis and restenosis in the graft vessel. MCP-1 is an indicator of early atherosclerosis, and its expression in blood vessels may promote infiltration of macrophages and induce proliferation of vascular endothelial cells [20]. In our study, the expression of MCP-1 in aortorenal vessels was also inhibited after islet transplantation, suggesting that microencapsulated islet transplantation was an effective strategy for inhibiting the development of graft vessels sclerosis after vascular reconstruction due to the improved control of blood glucose.

Damage to the glomerular filtration barrier in diabetic rats was ameliorated after islet transplantation. Impairment of the glomerular filtration barrier is a major cause of proteinuria in the early stages of diabetic nephropathy. The filtration barrier is mainly composed of endothelial cells, basement membrane, and epithelial cells (podocytes). Among these, podocytes are the most critical for the composition of the filtration membrane, and their impairment leads to proteinuria. It has been reported that the podocytes have a higher level of apoptosis, decrease in number, detach from the GBM, and are partially effaced at foot processes in the pathogenesis of DN [25–27]. However, the remaining podocytes failed to repopulate the depleted areas. As a consequence, the filtration membranes become more permeable, leading to proteinuria [28]. Another important structure of the filtration barrier, the GBM, is selectively permeable. As an extracellular matrix component of the glomerular filtration barrier, the GBM acts as a size-selective and charge-selective physical filter barrier [29]. At early stages of diabetic nephropathy, it is irregularly thickened, and the structure is thus impaired, allowing macromolecules to pass through [30]. In our study, the impairment of podocytes and GBM in diabetic nephropathy rats was alleviated in both the islet transplantation group and the combination group. This suggests that microencapsulated islet transplantation not only inhibits the atherosclerosis in the graft vessel but also alleviates damage to the glomerular filtration barrier in diabetic rats.

Islet transplantation is considered as an alternative therapy to the conventional insulin treatment and has been gradually accepted in recent years. It has been applied for clinical treatment on diabetic patients in some research institutions [9, 31]. Aortorenal bypass surgery is also thought of as an effective strategy in renal artery stenosis or occlusion to preserve renal function [32]. Thus, we developed a novel therapeutic strategy by combining MIT with revascularization of aortorenal bypass to control the blood glucose, restore the renal blood flow, inhibit atherosclerosis in the graft vessel, and alleviate damage in the glomerular filtration barrier. The combined strategy requires further investigation to confirm the beneficial effects for diabetic nephropathy patients with RAS.
